# Expression of EMT markers and mode of surgery are prognostic in phyllodes tumors of the breast

**DOI:** 10.18632/oncotarget.16497

**Published:** 2017-03-23

**Authors:** Xiaolong Feng, Lin Zhao, Honghong Shen, Xiaozhen Liu, Yang Yang, Shuhua Lv, Yun Niu

**Affiliations:** ^1^ Key Laboratory of Breast Cancer Prevention and Therapy (Ministry of Education), Tianjin Medical University Cancer Institute and Hospital, National Clinical Research Center for Cancer, Key Laboratory of Cancer Prevention and Therapy, Tianjin, China; ^2^ Department of Pathology, Tianjin Union Medical Center, Tianjin People's Hospital, Tianjin, China

**Keywords:** phyllodes tumors of the breast, epithelial to mesenchymal, mesenchymal stem cells, clinicopathological features, survival rate

## Abstract

Phyllodes tumors of the breast are rare neoplasms that account for <1% of all mammary tumors and 2-3% of fibro-epithelial neoplasms of the breast. We evaluated the clinicopathological characteristics of a cohort of 246 Chinese patients in relation to the expression of epithelial-to-mesenchymal (EMT) markers in benign, borderline and malignant tumors and the prognostic value of different surgical regimens. We observed that survival outcomes correlated with the mode of surgical management in the three patient groups. Expression of E-cadherin, Snail, Slug and Twist were higher in epithelial cells from borderline and malignant tumors than those in benign tumors, whereas the expression of N-cadherin was opposite. Levels of the EMT markers Snail and Slug in the stromal compartment increased with the advancing tumor grade. Expression of mesenchymal stem cell markers contributed to the inherent heterogeneity in the malignant tumors. Based on Cox models, surgical management emerged as an independent predictor for disease-free survival, whereas a history of recent growth and tumor grade were independent predictors for overall survival. These findings show that expression of EMT markers, the mode of surgical management, and a history of recent tumor growth had prognostic potential for patients with phyllodes tumors of the breast.

## INTRODUCTION

Phyllodes tumors (PT) of the breast are rare bidirectional neoplasms that represent <1% of all mammary tumors and 2% to 3% of fibroepithelial neoplasms of the breast and are typified by stromal proliferation [1–4]. The stromal changes are considered key during PT progression and the differences between the three stages of PTs has been widely debated over the last century. In 1838, Müller described the first case and called it “cystosarcoma phyllodes of the breast” [5]. More than half a century later, the term “cystosarcoma phyllodes” was replaced by “phyllodes tumor” to distinguish it from purely mesenchymal malignant tumors. During the 1970s, PT were classified into two categories, benign and malignant. In 1982, the World Health Organization adopted a new histopathologic classification of PT that included three categories, namely, benign, borderline, and malignant [6]. Although the histopathological type (benign, borderline or malignant) was an independent prognostic factor for survival, several other histological parameters that were prognostic factors in breast carcinomas were uncertain for PT. Further, although surgery is the standard treatment for PTs, the best type of surgery (local excision, breast-conserving surgery, total mastectomy or modified/radical mastectomy) has not been determined [1, 2, 7]. In the benign form of PT, wide local excision is usually preferred after taking the breast volume and tumor size into account. Total mastectomy (TM) is reserved to treat local recurrence (LR). However, the role of conservative surgery has not been clearly defined for borderline and malignant tumors. Also, the prognostic factors relevant to derive treatment options were limited by the low numbers of specimens available for studies.

The epithelial-mesenchymal transition (EMT) is a biological transformation process that enables transforming cells to acquire mesenchymal functions that include the ability of migration, invasiveness and elevated resistance to apoptosis [8]. Many studies regarding EMT have identified biomarkers for epithelial and mesenchymal cells such as E-cadherin, Vimentin, Snail, Twist and Slug [9–18]. All the grades of PTs showed local recurrence with the borderline and malignant forms showing the potential to metastasize to organs such as the lungs, bone and the liver. Therefore, the focus regarding metastasis of malignant PTs requires recognizing the stemness within the primary PTs similar to liposarcoma, leiomyosarcoma, rhabdomyosarcoma, osteogenic sarcoma or chondrosarcoma [19]. The multifunctionality of PTs demonstrating diverse sarcoma types is reminiscent of the mesenchymal stem cells (MSCs). Some studies showed that malignant PTs possess MSC-like properties and the mesenchymal biomarkers such as GD_2_ and ALDH_1_ that are highly expressed in mesenchymal stem cells [19]. Therefore, in this study of 246 patients, we explored the expression of EMT and MSCs biomarkers in different grades of PT (benign, borderline or malignant) with a goal to identify relevant prognostic factors and therapeutic options for benign, borderline and malignant PTs.

## RESULTS

### Clinicopathological parameters of patients

The clinical parameters of the 246 PT cases are summarized in Table 1. The 246 patients cases were sub-divided into three categories based on the histological type at diagnosis, namely, 42.3% benign (104 cases), 44.3% borderline (109 cases) and 13.4% malignant (33 cases). The 33 malignant tumor cases included five that were poorly differentiated with heterologous metaplasia. The age of the patients ranged from 11 to 76 years old (median: 46 years) at diagnosis. The median diameter of all phyllodes tumors was 6.0cm (range: 1.0-30cm). The median diameter was 4.5cm (range: 1.3-30cm) for benign tumors, 4.5cm (range: 1.0-28cm) for borderline tumors and 5cm (range: 1.5-18cm) for malignant tumors. At diagnosis, 77 (31.3%) patients were menopausal. Tumors in 145 (59%) patients were located in the left breast. 56 (23%) patients had recent tumor growth. 50 (20%) patients had experienced painful symptoms associated with the development of the disease. There was no statistical difference in age at surgery, tumor diameter, menopausal status, recent growth or painful symptoms between the three groups (*p*=0.498, *p*=0.345, *p*=0.903, *p*=0.706 and *p*=0.346, respectively). Of the 246 patients, 11 (4.5%) received breast-conserving surgery, 136 (55.3%) were treated by lumpectomy, 29 (11.8%) were treated by mastectomy and 70 (28.5%) were treated by modified or radical mastectomy. Significant differences existed between three groups according to different surgical managements (breast-conserving surgery, lumpectomy, mastectomy or modified/ radical mastectomy; P<0.001).

**Table 1:** Differences between tumor grade and clinicopathological parameters in 246 patients with phyllodes tumor of the breast (Values of P<0.05 were considered significant)

Parameters	Benign	Borderline	Malignant	χ^2^	*P* values
**Age at surgery (years)**					
≤50	73(70.2%)	70(64.2%)	20(60.6%)	1.4	0.498
>50	31(29.8%)	39(35.8%)	13(39.4%)		
**Tumor size (cm)**					
≤3	29(27.9%)	34(31.2%)	6(18.2%)	2.1	0.345
>3	75(72.1%)	75(68.8%)	27(81.8%)		
**Menopausal status**					
Yes	31(29.8%)	35(32.1%)	11(33.3%)	0.2	0.903
No	73(70.2%)	74(67.9%)	22(66.7%)		
**Recent growth**					
Yes	22(21.2%)	28(25.7%)	7(21.2%)	0.7	0.706
No	82(78.8%)	81(74.3%)	26 (78.8%)		
**Painful symptoms**					
Yes	20(19.2%)	21(19.3%)	10(30.3%)	2.1	0.346
No	84(80.8%)	88(80.7%)	23(69.7%)		
Surgical management*					
Breast-conserving surgery	5(4.8%)	6(5.5%)	0(0.0%)	49.3	<0.001
Lumpectomy	70(67.3%)	62(56.9%)	4(12.1%)		
Mastectomy	14(13.5%)	11(10.1%)	4(12.1%)		
Modified/radical mastectomy	15(14.4%)	30(27.5%)	25(75.8%)		

*P* values were calculated by Chi-square test.

* There were significant differences among benign, borderline and malignant phyllodes tumor of the breast in surgical management (χ^2^ =49.3, *P*<0.001).

### Immunohistochemistry of EMT biomarkers

**Figure 1:**
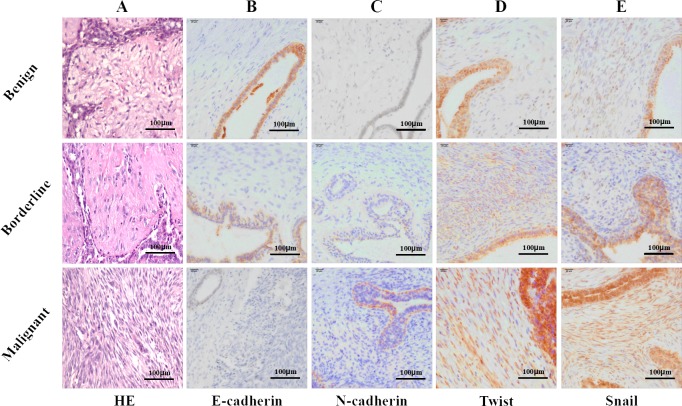
Representative photomicrographs of hematoxylin and eosin stain (H&E) and immunohistochemical stain (IHC) for E-cadherin, N-cadherin, Twist and Snail **(A)** H&E stained benign, borderline and malignant phyllodes tumor specimens (magnification × 200); **(B)** IHC showing E-cadherin expression in epithelial and stromal compartment (magnification × 400); **(C)** IHC showing N-cadherin expression in epithelial and stroma compartment (magnification × 400); **(D)** IHC showing Twist expression in epithelial and stroma component (magnification × 400); **(E)** IHC showing Snail expression in epithelial and stroma component (magnification × 400).

Expression of EMT markers was assessed using immunohistochemistry (IHC) in the 246 cases. Four cases (3 malignant PTs and one borderline case) were excluded from this study due to poor quality of the stained tissue section. IHC staining was separately scored for epithelial and stromal cells based on staining intensity and the percentage of positive cells. Stromal overgrowth was defined as absence of an epithelial component in at least one microscopic field at 40× total magnification (10×ocular objective and 4× microscopic lens objective). Excessive proliferation of stromal cells was a feature of borderline and malignant PTs. As shown in Figure 1, EMT markers expressed in at least 5% of cells were recognized as positive [19]. EMT markers were detected in the epithelial cells of 104 benign PTs with the following frequencies: E-cadherin (64.4%, 67/104), N-cadherin (45.2%, 47/104), Snail (34.6%, 36/104), Slug (31.7%, 33/104), Twist (34.6%, 36/104). Likewise, for the borderline (n=108) and malignant (n=30) PTs, the frequencies obtained were as follows: E-cadherin (48.1%, 52/108) and (43.3%, 13/30), N-cadherin (57.4%, 62/108) and (43.3%, 13/30), Snail (52.8%, 57/108) and (70.0%, 21/30), Slug (63.0%, 68/108) and (73.3%, 22/30), Twist (41.7%, 45/108) and (60.0%, 18/30). Whereas, the expression of E-cadherin, Snail, Slug and Twist in epithelial cells of benign PTs was lower than the borderline and malignant PTs, (*P*=0.025, *P*=0.001, *P*<0.001, *P*=0.044, respectively), no difference was observed for N-cadherin (*P* =0.146). For the EMT markers highlighting the positive stroma component, Snail (*P*=0.007) and Slug (*P*=0.001) increased with the advancing grade of PTs (Table 2).

**Table 2:** Differences between tumor grade and EMT markers in phyllodes tumor of the breast (Values of P<0.05 were considered significant)

Parameters	Benign	Borderline	Malignant	χ^2^	*P* values
**E-cadherin**					
epithelial *	67(64.4%)	52(48.1%)	13(43.3%)	7.4	0.025
stroma	33(31.7%)	32(29.6%)	10(33.3%)	0.2	0.906
**N-cadherin**					
epithelial	47(45.2%)	62(57.4%)	13(43.3%)	3.9	0.146
stroma	35(33.7%)	48(44.4%)	17(56.7%)	5.9	0.053
**Snail**					
epithelial*	36(34.6%)	57(52.8%)	21(70.0%)	14.2	0.001
stroma**	35(33.7%)	52(48.1%)	19(63.3%)	9.8	0.007
**Slug**					
epithelial*	33(31.7%)	68(63.0%)	22(73.3%)	27.6	<0.001
stroma**	33(31.7%)	58(53.7%)	19(63.3%)	14.7	0.001
**Twist**					
epithelial*	36(34.6%)	45(41.7%)	18(60.0%)	6.3	0.044
stroma	45(43.3%)	51(47.2%)	18(60.0%)	2.6	0.270

*P* values were calculated by Chi-square test.

* Immunohistochemical staining for EMT markers in the epithelial was analyzed by Chi-square test. There were significant differences among benign, borderline and malignant phyllodes tumor of the breast in E-cadherin, Snail, Slug and Twist-positive of epithelial cells (χ^2^ = 7.4, *P*=0.025; χ^2^ = 14.2, *P*=0.001; χ^2^ = 27.6, *P*<0.001; χ^2^ = 6.3, *P*=0.044).

^*^ Immunohistochemical staining for EMT markers in the stroma was analyzed by Chi-square test. There were significant differences among benign, borderline and malignant phyllodes tumor of the breast in Slug-positive of stroma cells (χ^2^ = 14.7, *P*=0.001).

### Expression of mesenchymal stem cells (MSCs) markers in malignant PTs

**Figure 2:**
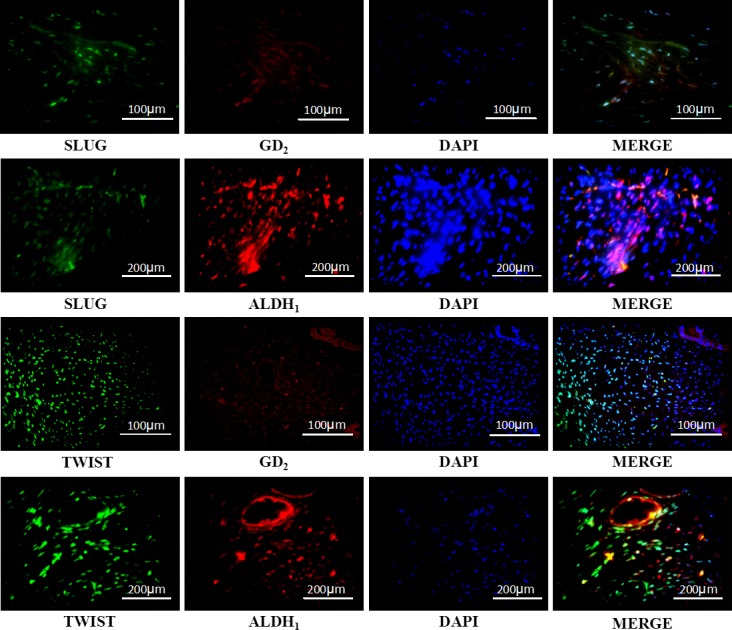
Representative photomicrographs of doubly stained immunofluorescence (IF) showing co-expression of EMT and MSCs markers in the malignant phyllodes tumor of the breast The photomicrographs show SLUG paired with GD2 or ALDH1 and Twist with GD2 or ALDH1.

To verify co-expression of MSC and EMT markers, 20 malignant cases were randomly picked for immunofluorescence. Co-expression of MSC (GD_2_ and ALDH_1_) and EMT markers was observed in 15 cases. Also, nuclear (Slug and Twist) and cytoplasmic (GD_2_ and ALDH_1_) staining was observed in these malignant PTs (Figure 2).

### Follow-up and survival analysis

**Figure 3:**
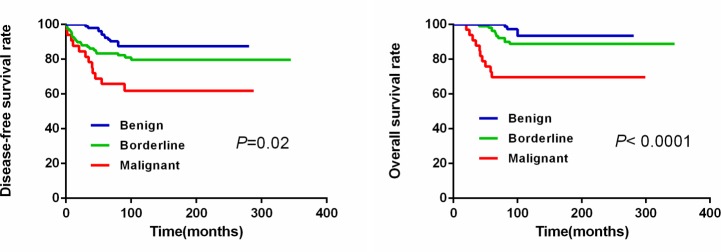
The 5-year DFS and 5-year OS curves in the three PTs patient groups

The median duration of follow-up was 102.5 months (range 60–345). During this time, 45 patients (18.3%) experienced recurrence and/or metastasis and 25 patients (10.2%) died. The most common focus of recurrence was the tumor bed and the most common metastasis was lung, followed by liver and brain. The rate of recurrence and metastasis in the malignant PTs group was 42.4% (14/33) compared to the borderline and benign groups that showed 26.6% (29/109), 13.5% (14/104), respectively (*P*= 0.001). Similarly, the overall survival was different among the three groups (*P*<0.01). Overall, the cohort of patients showed a 5-year DFS of 82.9% and a 5-year OS of 92.3%. The 5-year OS for the benign, borderline and the malignant groups was 97.1% (101/104), 96.3% (105/109) and 78.8% (26/33), respectively (*P*<0.001). The 5-year DFS for the three groups was 95.2% (99/104), 80.7% (88/109), 66.7% (22/33), respectively and was statistically significant (*P*<0.001; Table 3). Based on the Kaplan–Meier and log-rank statistic methods, we observed that the 5-year DFS and 5-year OS was significantly lower in the malignant group in comparison to the borderline and benign groups (Figure 3).

**Table 3:** Comparison of the recurrence and metastasis, 5-year DFS and 5-year OS in three groups (benign, borderline and malignant phyllodes tumor of the breast) (Values of *P*<0.05 were considered significant)

Parameters	Benign	Borderline	Malignant	χ^2^	*P* values
Local recurrenceor distantmetastasis*	14(13.5%)	29(26.6%)	14(42.4%)	13.1	0.001
5-year DFS*	99(95.2%)	88(80.7%)	22(66.7%)	18.7	<0.001
5-year OS*	101(97.1%)	105(96.3%)	26(78.8%)	17.2	<0.001

*P* values were calculated by Chi-square test.

* There were significant differences among benign, borderline and malignant phyllodes tumor of the breast in local recurrence or distant metastasis, 5-year DFS and 5-year OS (χ^2^ =13.1, *P*=0.001; χ^2^ =18.7, *P*<0.001; χ^2^ =17.2, *P*<0.001).

Based on univariate analysis (Table 4), advanced grade (*P*=0.01), Slug overexpression (*P*= 0.036) and surgical management (*P*<0.01) of PTs were the significant predictive factors for DFS. Only surgical management (*P*=0.009) was significant in the multivariate analysis. For OS, advanced grade at diagnosis (*P*<0.01), history of recent growth (*P*=0.007), Slug overexpression (*P*=0.028) and surgical management (*P*=0.002) of tumors were the significant predictive factors in univariate analysis, whereas, history of recent growth and grade of PTs were significant predictive factors in multivariate analysis (*P*=0.003 and 0.001). The rest of the parameters like age, tumor size, menopausal state, painful symptoms and expression of EMT markers (E-cadherin, N-cadherin, Snail, and Twist) were not statistically significant.

**Table 4:** Univariate analysis and multivariate analysis of malignant phyllodes tumor of the breast patients’ survivals (Values of *P*<0.05 were considered significant)

Parameters	DFS				OS			
	Univariate		Multivariable		Univariate		Multivariable	
	5-year-DFS %	*P*	95%CI	*P*	5-year-OS %	*P*	95%CI	*P*
**Age**								
≤50	82.5%	0.509			89.5%	0.500		
>50	81.1%				90.2%			
**Size**								
≤3	88.2%	0.106			95.6%	0.078		
>3	79.1%				87.6%			
**Menopausal**								
Yes	79.2%	0.483			87.0%	0.339		
No	82.2%				91.1%			
**Growth**							1.58-9.15	0.003*
Yes	83.6%	0.135			80.7%	0.007*		
No	75.4%				92.6%			
**Painful**								
Yes	83.6%	0.137			86.0%	0.374		
No	74%				90.8%			
**Grades**			0.90-2.23	0.131			1.62-7.50	0.001*
Benign	88.5%	0.001*			96.2%	<0.001*		
Borderline	80.7%				89.9%			
Malignant	63.6%				69.7%			
**EMT marker**								
E-cadherin	82.7%	0.656			91.7%	0.281		
N-cadherin	81.5%	0.925			90.3%	0.830		
Snail	78.6%	0.199			88.0%	0.352		
Slug	76.6%	0.036*	0.74-2.64	0.304	85.5%	0.028*	0.59-3.56	0.421
Twist	78.2%	0.227			88.1%	0.391		
**Surgical management**			1.08-1.74	0.009*			0.80-1.57	0.518
Breast-conserving surgery	100%	<0.001*			90.9%	0.002*		
Lumpectomy	89.6%				95.6%			
Mastectomy	64.3%				82.1%			
Modified/radical mastectomy	71.4%				82.9%			

DFS, disease-free survival; OS, overall survival.

## DISCUSSION

Phyllodes tumors are uncommon fibroepithelial tumors of breast that are difficult to pre-operatively diagnose with unpredictable clinical outcomes [6]. Although the biomarkers of PTs have been investigated previously, reliable biomarkers for clinical diagnosis and adjuvant therapies remain unclear [20, 21]. The invasive and metastatic stages of breast cancer progression are closely linked to the epithelial–mesenchymal transition (EMT) that results in the transformed cells acquiring the abilities of invasiveness, migratory and stemness [11, 22–24]. In our study, we hypothesized that the EMT process was associated with PTs progression and therefore analyzed the status of the EMT markers in different stages of the tumors in the 246 clinical specimens. We demonstrated overexpression of key EMT proteins, namely, E-cadherin, N-cadherin, Snail, Slug and Twist in PTs. We also observed that increased E-cadherin, Snail, Slug and Twist expression was associated with the malignant phenotype of PTs. Our findings were concordant with previous studies that showed that EMT markers (E-cadherin, Snail, Slug and Twist) expression (*P*=0.025, *P*=0.001, *P*<0.01, *P*=0.044, respectively) correlated more strongly with borderline and malignant PTs than with benign PT [25]. However, no significant difference in N-cadherin (*P*=0.146) expression was observed between the malignant/borderline and the benign groups. Similarly, in stromal cells, Snail (*P*=0.07) and Slug (*P*=0.01) overexpression correlated with the increasing grades of PTs.

The clinicopathological features of the malignant PT patients showed the largest rate of modified or radical mastectomy. In spite of extensive resection, the malignant patients had most local recurrences and/or metastasis accounting for the worst five-year DFS and OS. It was observed that the grade of PTs influenced their distinct biological behaviour [26]. Tse and colleagues showed that the rate of recurrence increased as the grades progressed and ranged from 10 to 25% for benign to 32% for borderline and about 40% for malignant cases [26]. In our study, there was a 13.5%, 26.6% and 42.4% local recurrence and/or metastasis in the three groups with longer follow-up. As expected, the malignant group had poorer prognosis than the borderline and benign groups. Compared to malignant epithelial neoplasm of breast and true sarcomatous tumors, the malignant phyllodes tumors were not as lethal. A former epidemiologic review showed that malignant PTs had a 5-year survival rate of 80% after surgery [27]. Our results were consistent with the previous reports with a 5-year-OS of 78.8% and 5-year-DFS of 66.7%. Clinicopathological factors like the grade of tumors, mode of surgery and Slug expression influenced patient survival.

We further evaluated expression of EMT markers in epithelial and stromal cells of the PTs specimens. We observed stronger E-cadherin expression in the epithelial compartment in comparison to the stroma. Among the 242 cases of PTs, high expression of E-cadherin was observed in the epithelial compartment of 132 cases (64.4% of benign, 48.1% of borderline, and 31.1% of malignant cases), whereas, only 75 cases (31.7% of benign, 29.6% of borderline, and 33.3% of malignant) showed high expression in the stroma. In addition, high expression of E-cadherin in the epithelial compartment was more frequent in benign PTs compared with malignant and borderline PTs (*P*=0.025). However, the E-cadherin expression was not different in the stroma of the different grades (*P*=0.906). High expression of Snail with a nuclear staining pattern was observed in both the epithelial and the stromal cells of PTs tissues. The high expression of Snail in the epithelial and stromal cells was significantly different in three groups (*P*=0.001 and *P*=0.007). Similarly, Slug and Twist showed high expression and a nuclear staining pattern in the epithelial and stromal cells of PTs with significant differences with progressive grades. Furthermore, Slug expression associated with the 5-year-DFS and 5-year-OS of the patients.

MSCs play an important role in the progression of malignant tumors. Lin and colleagues demonstrated that ALDH_1_ and/or GD_2_ markers could be used for cancer stem cells research in malignant PTs [19]. We found co-expression of EMT and MSCs markers in 75% of the malignant cases (15/20 cases). Therefore, we hypothesized that malignant PTs possessed MSC characteristics and differentiated towards soft tissue tumor similar to liposarcoma, leiomyosarcoma, rhabdomyosarcoma, osteogenic sarcoma or chondrosarcoma. Further analysis with increased number of malignant PTs specimens is necessary to understand the significance of the co-expression and other statistics.

Surgery remains the only primary treatment in patients with PTs [28]. We evaluated survival outcomes of the 246 PTs patients, of which, 136 (55.3%) were treated with Lumpectomy, 11 (4.5%) with BCS (Breast-conserving surgery), 29 (11.8%) with mastectomy and 70 (28.5%) with modified or radical mastectomy. Our studies found that the mode and extent of surgery associated with the 5-year-DFS (*P*<0.01) and 5-year-OS (*P*=0.002) rates. Previous studies had shown no significant differences in survival between patients that underwent BCS versus mastectomy and our data were partially concordant [29].

In conclusion, our study showed that expression of some EMT markers increased with the progression of tumor grade could be of prognostic value. We also observed that the mode of surgery, the grade of tumor and history of recent tumor growth were independent predictors for decreased OS. This study also indicated that PTs had a relatively milder prognosis compared to other epithelial tumors of the breast. The EMT signaling pathway and the mesenchymal stemness of PTs tumors present novel therapeutic targets in the future.

## MATERIALS AND METHODS

### Patients and tissue samples

This study was approved by the Tianjin Medical University Cancer Institute and Hospital, China and was performed in accordance with the ethical standards laid down by the 1964 Helsinki Declaration and its later amendments. After analyzing the clinicopathological parameters of breast cancer patients at the Cancer Hospital of Tianjin Medical University from April 1987 to June 2010, 246 patients ranging from 11 to 76 years old with a median age of 46 years were selected for our study. The inclusion criteria were: (a) The patients were diagnosed with PTs; (b) The patients had undergone surgery; (c) The patients had no previous neoadjuvant systemic therapy and (d) They completed the follow-up study period. Two senior pathologists reviewed the diagnosis and classification of all forms of PTs based on the 2012 WHO Classification of Tumors of the Breast by using hematoxylin and eosin (H&E)-stained sections of samples from each selected case. The expression of EMT and mesenchymal stem cell biomarkers were evaluated for all 246 cases by performing IHC. To study the clinical relevance of EMT biomarkers expression, the 246 cases were divided into three groups (104 benign cases, 109 borderline cases, and 33 malignant cases) according to their tumor grade.

### Pathological review

All cases were reviewed by two senior pathologists (Niu Y. and Lv SH.). Tumors were evaluated for stromal cellularity and stromal nuclear atypia [30]. The stromal cellularity was classified as (a) low : if the stromal cells were sparsely distributed and appeared as slender spindle cells; (b) moderate : if the cellularity was moderate and the stromal cells were similar to spindle cells with slight pleomorphism and (c) high, if the cellularity was high with the stromal cells showing back-to-back arrangement with malignant appearance and nuclear pleomorphism. When the cellularity within an individual tumor was heterogeneous and showed other sarcoma of soft connective, the higher score was recorded (Figure 1). The stromal mitotic activity was quantified based on ten high-power fields (HPFs) in the most mitotically active areas of the stroma. Heterologous differentiation components such as rhabdomyosarcoma, chondrosarcoma and osteosarcoma were screened by hematoxylin–eosin staining and confirmed by additional immunohistochemical stains such as Myogenin, Myo D1, Desmin and S-100 protein.

According to 2012 WHO Classification of Tumors of the Breast [31], the histopathological grade of PTs was determined as low, moderate or high grade cellularity with nuclear atypia and classified as benign, borderline and malignant, respectively. Moreover, cellularity and/or mitotic counts were also considered. The 2012 WHO Classification of Tumors of the Breast proposed a cutoff of 5 mitoses per 10 HPFs to distinguish between benign and borderline PT. Moderately atypical cases with both ≥ 10 mitoses/10HPFs and diffusely high cellularity were up-graded to malignant. In summary, malignant, borderline and benign PTs showed ≥ 10, ≤ 9 and ≤ 4 mitoses per 10HPFs, respectively.

### Immunohistochemical analysis

The tissue samples were fixed in 10% neutral buffered formalin and then paraffin-embedded. Then, 3μm sections were cut and de-waxed on coated slides with xylene followed by rehydration with distilled water. Further, sections were treated with citrate buffer solution (pH 6.0) or ethylene diamine tetraacetic acid (EDTA) buffer (pH 8.0) for 2.5 min in a pressure cooker for antigen retrieval. This was followed by treatment with 3% hydrogen peroxide and blocking in normal goat serum for 20 min. The primary antibodies that were used in this study were: E-cadherin (ZSGB, ZM-0565, CHN), N-cadherin (ZSGB, ZM-0094, CHN), Vimentin (ZSGB, ZM-0511, CHN), Snail (Abcam, ab180714, UK), Slug (Abcam, ab27568, UK), Twist (Abcam, ab50581, UK), GD_2_ (Abcam, ab68456, UK) and ALDH_1_ (Abcam, ab56777, UK). They were used at dilutions of 1:150, 1:50, 1:150, 1:100, 1:100, 1:100, 1:100 and 1:70, respectively. After incubation with primary antibodies at 4°C overnight, the secondary biotinylated goat anti-mouse/anti-rabbit immunoglobulin was applied for 20 min followed by incubation with peroxidase-conjugated streptavidin. Then, sections were incubated with 3, 3′-diaminobenzidine tetrahydrochloride (DAB) and counterstained with hematoxylin. Sections of normal breast epithelial cells and external tissues were processed simultaneously and served as positive controls. For negative controls, primary antibodies were replaced by PBS. The stromal and epithelial cells of the tumors were scored separately based on IHC staining by two senior pathologists (Niu Y. and Lv SH.) based on staining intensity (negative, weak, mild or strong), percentage of positive cells and the cellular localization of staining (nucleus, cytoplasmic or membranous) of these slides.

### Immunofluorescence staining

The paraffin sections of malignant PTs were dewaxed in xylene and rehydrated followed by antigen retrieval in ethylene diamine tetraacetic acid (EDTA) buffer (pH 8.0) by pressure cooking for 2 min. Subsequently, the sections were permeabilized with 0.1% Triton X-100 and incubated overnight at 4°C with rabbit anti-human Snail (Abcam, ab180714, UK), Slug (Abcam, ab180714, UK), Twist (Abcam, ab50581, UK), mouse anti-human GD_2_ (Abcam, ab68456, UK) and ALDH1 (Abcam, ab56777, UK) antibodies. Then, the sections were rinsed and incubated with the secondary antibody conjugated to the fluorescent dyes, Alexa Fluor® 488 and Alexa Fluor® 594. Finally, the sections were rinsed and incubated with 4′, 6-diamidino-2-phenylindole dihydrochloride (DAPI; Life Technologies, USA) and visualized under a fluorescence microscope.

### Follow-up study

The cases were followed-up until March 1, 2016 and ranged from 60 to 345 months with a median duration of 102.5 months. Detailed dates of follow-up cases were collected via medical records, telephone calls and a study questionnaire every 3 months for the first 2 years and every 6 months for the next 3 years. Disease-free survival (DFS) was defined from the date of surgery to the date of local relapse and/or distant metastasis. Reappearance of tumor in the treated remnant breast, skin or chest wall was defined as local recurrence. Distant metastasis was considered when the tumor metastasized at a distant site like lungs, bone and/or liver.

### Statistical analysis

Statistical analyses were performed using SPSS 19.0 software (SPSS Inc., Chicago, IL, USA). Univariate χ^2^ tests were used to investigate associations between tumor characteristics and biomarker positivity. When necessary, fisher's exact test was used. Survival analysis was performed by using the Kaplan–Meier curves and log-rank tests. Cox proportional hazards modeling for multivariate analysis was used to identify variables of independent prognostic value. Results were considered significant if a *P* value of<0.05 was obtained.
